# Are teachers techno-optimists or techno-pessimists? A pilot comparative among teachers in Bolivia, Brazil, the Dominican Republic, Ecuador, Finland, Poland, Turkey, and Uruguay

**DOI:** 10.1007/s10639-020-10380-4

**Published:** 2020-11-11

**Authors:** Łukasz Tomczyk, Vladimir Costas Jáuregui, Cibelle Albuquerque de La Higuera Amato, Darwin Muñoz, Magali Arteaga, Solomon Sunday Oyelere, Özgür Yaşar Akyar, Mariana Porta

**Affiliations:** 1 Pedagogical University of Cracow, Kraków, Poland; 2 Universidad Mayor de San Simón, Cochabamba, Bolivia; 3 Mackenzie Presbyterian University, São Paulo, SP 01302-907 Brazil; 4 Universidad Federico Henriquez y Carvajal (UFHEC), 11005 Santo Domingo, Dominican Republic; 5 Universidad del Azuay, Cuenca, Ecuador; 6 University of Eastern Finland, Yliopistonranta 1, 70210 Kuopio, Finland; 7 Hacettepe University, Ankara, Turkey; 8 CENUR Noreste, UDELAR, Montevideo, Uruguay

**Keywords:** attitudes towards new media, restrictions on the use of smartphones in schools, experiences with e-learning, self-evaluation of digital competence, comparative education, teachers, schools, internet, New media usage style

## Abstract

The aim of the article is to highlight the key elements related to the implementation of new technologies in education from the perspective of the opinions and experiences of educators in the field in Bolivia, Brazil, the Dominican Republic, Ecuador, Finland, Poland, Turkey, and Uruguay. The text compares issues related to attitudes towards the use of new media in education, experiences with different forms of e-learning, and the level of restrictions on the use of smartphones in school. These variables are juxtaposed with the self-assessment of digital competence and how cyberspace is used. The survey was conducted using a standardised survey questionnaire translated into the relevant national languages in the first half of 2019, and involved a sample of 873 teachers representing eight countries. On the basis of the pilot studies it was noted that: 1) Teachers from LAC and EU like to use digital media - this is a constant trend independent of geographical location; 2) Teachers note that new technologies are not always better than analogue didactic aids; 3) Teachers from selected countries (the Dominican Republic, Brazil, Turkey, and Uruguay) have much greater techno-optimism in themselves than teachers from Bolivia, Poland, Finland and Turkey in terms of the impact of ICT on student motivation and engagement; 4) In all countries teachers prefer free online courses (the different forms of e-learning are used most often by those in the Dominican Republic, and the least often in Bolivia and Poland); 5) In each country teachers who highly value their own digital competences and have a positive attitude towards new media use ICT much more actively; 6) There is also a global trend in that the extensive use of cyberspace (typical e-services) appears in combination with the extensive use of various forms of e-learning; 7) Teachers from Ecuador are most likely to want to ban the use of smartphones in schools. The most liberal approach in this respect is taken by the Uruguayans; 8) The knowledge of the conditions related to restricting the use of smartphones goes beyond the analyses related to the style of use and attitude towards new media. This article is the result of pilot studies conducted within the framework of the SMART ECOSYSTEM FOR LEARNING AND INCLUSION project carried out in selected Latin American, Caribbean (LAC) and European (EU) countries.

## Introduction

In the world before Covid-19, the role of digital media in education was already increasing in scope, leading to an increase in discussions about the consequences of this shift towards the digital. With the global crisis provoked by the Covid-19 pandemic, these discussions increased and the importance of fully integrating digital technologies and education became more clearly evident. The social distancing imposed by this public health crisis exposed the gaps in digital education, especially in teaching-learning environments. In that sense, the use of ICTs in education seeks to complement, enrich and positively transform the learning environment. In addition, the increasing number of technological platforms available has encouraged people to transfer aspects of their lives, whether social, professional, or cultural, into these spaces, thus providing opportunities related to the democratization of education and social inclusion (Foulger et al. 2017).

The use of technology makes it possible to reduce physical or contextual distances such as relate to disability or socioeconomic disadvantage. However, this technological immersion requires from its users a specific set of competencies, skills and abilities in order to face present demands and, also, to achieve a better exercise of rights. In this perspective, the teacher stands out as the mediator of the teaching-learning process, and can be considered as a person who needs to mediate both aspects of technology and the learning process. Hence it is essential that teachers acquire digital skills that allow them to develop and transmit their knowledge in this, the digital age. (Bond et al. 2018).

Consequently, the purpose of this study is to understand the teacher’s perception of new media, restrictions on the use of smartphones in schools, experiences with e-learning, and the self-assessment of digital competence among educators in different countries. The countries involved in this study have different socio-cultural contexts, and these strengthen the relevance of the research considering the possibility of significantly expanding our understanding of the variables and their effects based on the diversity of each country and the perception of its teachers.

The key point for this study is to understand how teachers perceive technological resources for education. This knowledge, based on the specific characteristics of the different countries involved, will allow further reflection on the redesign of the technological reality for teaching and learning. This restructuring articulates digital, pedagogical and social relationships, expanding the possibilities of democratizing digital education while respecting diversity.

The professional development of teachers is of fundamental importance in understanding the effects of the expansion and incorporation of technologies and digital media in educational systems, institutions and practices. It is also important to highlight institutional support and the proposal of development strategies aimed at the teaching professional as aspects of crucial relevance. Only with this support will teachers be able to face the challenges of incorporating technology into the teaching-learning environment. (Tondeur et al. 2017).

All things considered, this study is necessary to assess the effects and consequences of the expansion and incorporation of digital technologies and media in educational systems, institutions and practices based on the perception of one of the chief actors in the process, the teacher. In this sense, we seek to move towards critical and innovative pedagogical practices, continuing the research developed by the group of researchers involved in the SMART ECOSYSTEM FOR LEARNING AND INCLUSION project carried out in selected Latin American, Caribbean (LAC) and European (EU) countries. With the results of the study, it is intended to advance the understanding of teachers’ perceptions from the different countries involved, and to expand the reflections on pedagogical strategies and practices for teaching in digital environments. It is also intended to evaluate the processes of the appropriation of digital technologies by teachers and students, creating open educational resources and proposals for inclusive and accessible education environments.

## Theoretical framework

This study is underpinned by two related frameworks. The technology acceptance model, first proposed by Davis (1989), is comprised of the core variables of user motivation and outcome variables. The study also makes use of Technology Pedagogy Content Knowledge developed by Koehler and Mishra (2009). Through the merging of the subdomains of the two theoretical frameworks technology from TPCK and by adopting user motivation and outcome variables in our context we focused on four variables: attitude to new media, the way in which new media are used, experiences with e-learning, and self-evaluation of digital competence.

A previous study carried out among pedagogy students on attitudes toward new media led to the delineation of four categories of attitude to new media: techno-optimist, techno-realist, techno-pessimist, and techno-ignorant (Tomczyk et al. 2017). According to the authors, techno-optimism is characterized by an enthusiastic attitude towards new media, where new media are understood as the source of positively-evaluated transformations in the life conditions of modern man, and which have a positive influence on the quality and effectiveness of education. The techno-realist is characterized by a certain distance as regards new technologies, though this does not mean a reluctance to modify their own style of working according to technological progress. Instead, it involves a careful, conscious openness to the new possibilities new media present. The techno-pessimist is characterized by a negative attitude towards new technologies and a belief that they lack usefulness (as a moderate option) or unfavorable for human development and functioning (as a radical option). The techno-ignorant is characterized by a lack of involvement in learning about new media.

In terms of how new media are used we explore the different ways employed by teachers when it comes to the use of new media such as publishing messages on the Internet, consuming Internet streaming (eg. VOD), creating video, using a file sharing service, participating as a member of a group, accessing online services – e-government, buying/selling goods, and engaging in leisure activities.

The teacher’s experiences with e-learning is another variable, and we explored studying on an obligatory online course, taking free e-learning courses, taking paid online courses, and participating in online study groups.

The self-evaluation of digital competence can be considered as one’s own perspective about the ability to use digital tools and services. In this regard, we explored competences such as using a text editor (e.g. Word, writer), using a spreadsheet program (e.g. Excel, Calc), using a presentation program (e.g. Power Point, impress), using a graphics program (e.g. Picasa, Gimp), and knowledge about the dangers of the digital world (e.g. cyberbullying, Internet addiction, sexting).

## Previous research findings

Around the 1990s a movement to use technology in education was set up, and as has happened to many other fields, the application of such technologies sought to increase both efficiency and effectiveness in education. Implementing technologies in education is a many-sided process with different aspects connected to pedagogy, knowledge, attitudes, content, and perceptions among others.

Globally, technology looks attractive for many purposes; in education, the possibility that ICT provision would make instruction more equitable has put teachers, curriculum designers and authorities under pressure to create and implement “a go-to technology”. In the case of Latin America, the introduction of ICTs into education has come from an initiative of the Ministries of Education. According to Castillo-Valenzuela and Garrido-Miranda (2018), this initiative happened not only to make education more equitable, but to reduce the differences cause by the digital divide. Among the countries that pioneered this work, there is Brazil with the National Programme for Educational Technology, ProInfo. Then a second wave of programs appeared in Latin America, mainly seeking to transform the teaching and learning processes and to distribute equipment to students and schools. Such is the case with Uruguay’s *Plan Ceibal* and the *Uso de tablets en el aula* in Ecuador (Ponce and Rosales 2018). Although there have been many attempts in this region to implement ICTs in education, there are still areas that need further analysis, from basic access to electricity and access to technology at one end of the spectrum to teacher training on the use and application of technologies in the classroom at the other (Lugo and Brito 2015).

As education is a double sided entry, both teachers and students play an important role in the implementation of ICTs in education. However, it is the teachers who decide upon the activities, content and strategies employed to reach a predetermined learning outcome. Thus, the implementation of technology in classrooms depends on how confident the teacher feels when using technological tools in class. The teacher’s attitude towards the relevant devices determines the success or failure of the implementation of ICT implementation in the classroom. If a teacher shows a positive attitude, there is a greater chance of success. However, teachers may also feel undermined by the use of ICTs, and in this case there would be a decrease in the use of ICTs in class (Unser 2017).

ICTs offer excellent solutions for different problems that arise in a class. Oyelere and Tomczyk (2020) talk about how there is a module aimed at games, there are online libraries and there are many other digital strategies that enhance the process of learning. However, technology alone cannot solve every problem in the classroom. The tools don’t drive the teaching, but they do demand changes to the way we teach. As Gibson (2016) points out, the road of teaching is composed of both teaching styles and technology styles. The different weight a teacher gives to each style will mark a teacher-centered class or a student-centered class. According to Brush and Saye (2009), this problem arises because pre-service teachers are taught to develop their technology skills rather than to integrate technology and teaching.

The changes happening in Latin American and Caribbean societies have led to changes in education too. ICTs in education generate confidence in governments and authorities to enhance the learning process for students. However, as mentioned by Ames (2015), the use of technology in education should not be an isolated application. Its adoption in education should go hand in hand with the sociocultural environment, so that aspects such as infrastructure, application, maintenance and technical support are offered to schools. Training about software creation and digital content should be highlighted for its positive impact on education. Otherwise, teachers’ negative experiences may lead to a lack of confidence in the use of ICTs in classrooms.

According to Arteaga et al. (2020), governments have placed a lot of emphasis on preparing pre-service teachers in the use of ICTs in their classrooms in many countries in Europe, Latin America and the Caribbean. This training usually begins in their college programs as a way to facilitate the educational programs offered by these governments. Although the efforts have been great, the teachers themselves need to feel comfortable when using technology in their classes, so the training should aim at developing self-confidence in those abilities. Habibi et al. (2018) states that “the success of any initiatives in implementing technology in an educational program depends on the supports and attitudes of the involved users” (p. 46).

Access to Massive Open Online Courses’, on the other hand, are changing the way teachers and pre-service teachers view education. Courses delivered by Web 2.0 tools include video conferencing with teachers and professors all over the world. According to Culquichicón et al. (2017) these courses have become so popular due to the implementation of new educational techniques, the schedule on offer, and the “absence of geographical barriers” (p. 3) facilitated by different platforms such as Coursera and edX, among others.

Smart education or smart-environment education is growing rapidly due to the huge amount of material available online. Abdurazakov et al. (2016) consider cyberspace as a means for enhancing interaction in the virtual world. This new world then expands values, knowledge and culture. Thus both teachers and students can interact with texts or any other kind of resource through the net. Learning how to adapt such material for use in class requires not only good knowledge of the subject but a sound knowledge of the students’ context. Pedagogical criteria should be appropriately applied in the creation of online education, so that healthy relationships between the stakeholders in the education process are well established.

Computer-based platforms are not the sole means of applying ICTs to education. The smartphone represents a great source of technology in the classroom. This device has caused a lot of fuss among teachers, schools, and parents. On the one hand, parents are reluctant to encourage their children to use their smartphone in class. Many reasons are given, but the most common are social and economic factors, while pedagogical reasons are not core parental resistance (Hadad et al. 2020). Wiederhold (2019) says that although studies have shown a reduction in cognitive function connected with the use of a smartphone in class, most younger children do still bring technological devices to school. This excessive use could be the reason for why children are maturing faster, but the access they have to social media through these devices causes a great distraction and can lead to depression. For this reason, many teachers are calling for a ban on smartphones, while others suggest that their use should only be moderate.

## Methodology

### Research purpose and research problems

The aim of the research was to compare the declarations of teachers from three European countries (Finland, Poland, and Turkey) and five from Latin America and the Caribbean (Bolivia, Brazil, the Dominican Republic Ecuador, and Uruguay) on key elements related to the use of new digital technologies in learning, teaching. The research was carried out in the framework of the project SMART ECOSYSTEM FOR LEARNING AND INCLUSION - ERANET17/ICT-0076 SELI, which seeks to diagnose the conditions related to the use of digital media in education, as well as to design an e-learning platform for education, lifelong learning and the development of an optimal learning environment regardless of age or deficit. Taking into account the atypicality of the assumed research goal, including the rarity of research used to compare features among pedagogical staff, as well as the scope of modern media pedagogy, the research problems were narrowed down to the following questions:

- RQ1 - What attitudes towards new technologies do teachers in selected LAC and EU countries possess?
- RQ2 - What is the relationship between the attitude to new media in education and experiences with e-learning, how new media is used and self-assessment of digital competence?
- RQ3 - What are the teachers’ opinions on restricting the use of smartphones in schools?
- RQ4 – To what extent are attitudes towards the use of smart phones at school evolving with media attitudes, learning experiences and how new media is used?

### Research tool

The research tool was designed in cooperation with an interdisciplinary and international team consisting of educators, computer scientists, and sociologists. The tool was originally developed in English and then translated into the relevant national languages (Spanish, Portuguese, Polish, and Turkish). The tool has been adapted to the cultural conditions of each country and has been evaluated by pilot studies and external experts (Oyelere and Tomczyk 2020).

The final version of the diagnostic questionnaire consisted of a triangulation of research tools with internal consistency Cronbach’s Alpha = 0.897. The whole tool was checked by Exploratory Factor Analysis (EFA). Based on this EFA, the collected data can be divided between four factors. Each of the factors groups indicators of one of the variables. The tool has the following characteristics (Bartlett’s test Χ^2^ = 11,219.172, df = 325.000, *p* < .001; Kaiser-Meyer-Olkin test = 0.910) (the detailed factor loading level is in Appendix Table 6). The tool consists of four main variables:

- Attitude to new media (8 indicators: I like to use digital technology, Digital technologies have positively changed our lives, It is necessary to use digital technologies in the process of learning and teaching, Websites are useful for teaching and learning, Digital teaching aids are better than physical teaching aids for improving learning, The teacher’s use of digital technologies has a positive impact on student learning, The teacher’s use of digital technologies has a positive effect on student motivation). Responses were given on a 5-step Likert scale (1- I strongly disagree, 5- I strongly agree). The tool had the following internal consistency: Cronbach’s Alpha = 0.900.
- How new media is used (8 indicators: Publishing messages on Internet, Consuming Internet streaming services (eg. VOD), Creating video, Using a file sharing service, Participating as a member of a group, Accessing online services – e-government, Buying/Selling goods, Leisure activities). The respondents gave answers that characterised the frequency of their use of digital media in terms of typical activities undertaken in cyberspace on a five-stage Likert scale from 1 - never to 5 - very often. The tool was characterized by the following internal consistency properties: Cronbach’s Alpha = 0.863
- Experiences with e-learning (4 indicators including: Study in an obligatory online course in my career or in my postgraduate studies, Taking free e-learning courses (online courses - e.g. language, ICT), Taking paid online courses, Participating in online study groups.) Respondents were invited to give answers on a 5-degree Likert scale, from 1 - never to 5 - very often). The tool had the following internal consistency properties: Cronbach’s Alpha = 0.850
- Self-evaluation of digital competence (containing 5 indicators: Using a text editor (e.g. Word, writer), Using a spreadsheet program (e.g. Excel, Calc), Using a presentation program (e.g. Power Point, impress), Using a graphics program (e.g. Picasa, Gimp), Knowledge about the dangers of the digital world (e.g. cyberbullying, Internet addiction, sexting)). Each of the teachers had the opportunity to assess their own knowledge and support the self-assessment of the components of digital competence on a Likert scale from 1 - very low to 5 - very high. The internal consistency of the variable was Cronbach’s Alpha = 0.845

### Research procedure

The study was carried out in Bolivia, Brazil, Ecuador, Finland, Poland, the Dominican Republic, Turkey and Uruguay by the teams of researchers participating in the SELI Project. Each country worked with two or more researchers to develop the surveys and in the subsequent analysis of the responses. The study was carried out from June to October 2019; The duration of the study depended on access to teachers according to the peculiarities of term times (for winter, summer and end of the school period) managed by the government education organizations of each country. Thus, in each country, the study lasted between a fortnight and four months. The research was supported by the following agencies: Bolivia: Ministerio de Educación – Vice Ministerio de Ciencia y Tecnología, MINEDU; Brazil: Fundação de Amparo à Pesquisa do Estado de São Paulo, FAPESP; Dominican Republic: Ministerio de Educación Superior, Ciencia y Tecnología, MESCyT; Ecuador: Secretaría de Educación Superior, Ciencia, Tecnología e Innovación, SENESCYT; Finland: Academy of Finland, AKA, Research Council for Culture and Society; Poland: Narodowe Centrum Badań i Rozwoju, NCBiR; Turkey: Turkiye Bilimsel vê Teknolojik Arastirma Kurumu, TUBITAK; Uruguay: Agencia Nacional de Investigación e Innovación, ANII.

The data collection process for each country is briefly described below:

In Bolivia, three university professors carried out the study in June 2019. The initial survey was planned using Google Forms; unfortunately, no teachers responded to the electronic survey sent by the social networks of the Ministry of Education. Due to the lack of a response by electronic means, the team non-randomly selected several schools in the urban area among public, private and ‘agreement’ schools (the last of these are involved in the special education of children with physical and cognitive disabilities) to be surveyed by printed means, which were automatically tabulated using OCR; then the tabulation was randomly verified, and the tabulation of any forms not recognized by the OCR device was completed by hand. Finally, the team worked on the analysis of the data obtained.

The Brazilian research team, made up of four university professors, carried out the study from August to September 2019. The data collection was channelled electronically, by sharing a Google Form through social networks and email. The teachers’ culture of sharing was relied upon to expand the number of teachers who received the questionnaire.

The Dominican Republic’s research team applied a digital form (Google Form) both in person and disseminated on the web. The work for the study was carried out from July to August 2019.

The Ecuadorian research team conducted the study from July to October 2019. The data collection was through the web, in the form of a voluntary survey for teachers in Ecuador.

Researchers from Finland recruited respondents by submitting a link to the questionnaire on a Google Form. The study was carried out between June and September 2019.

In Poland, the study was carried out by three experts in media pedagogy during June 2019. Printed forms were used in surveying the teachers. The teachers who participated in the survey were chosen non-randomly by previous work contacts with the Pedagogical University of Krakow.

Three experts made up the team from Turkey; the team is part of the department of sports teaching and physical education of the faculty of sports science. The study used mixed printed and Google Form surveys filled out voluntarily by the physical education and sports teachers.

The Uruguayan research team, with experience in the areas of education and technology, carried out the study in June 2019 among teachers of higher education centres in Uruguay. A Google Form was circulated in a non-random way using the snowball technique. The study areas were the south and northeast of the country.

### Characteristics of the research sample

Below is a tabular sociodemographic description of the sample of teachers examined in the specific countries (see Table 1).

**Table 1:** Sociodemographic characteristics

	Bolivia	Brazil	Dominican Republic	Ecuador	Finland	Poland	Turkey	Uruguay
N	137	104	145	145	88	106	87	107
**Age** Mean (SD)	46	42.59 (11.76)	42.81 (10.96)	43.93 (9.30)	37.45 (6.49)	37.84 (10.07)	37.97 (7.5)	46.41 (10.01)
Gender								
Female	66%	59.61%	53%	55%	41%	84%	36.8%	74%
Male	33%	40.39%	47%	44%	54%	16%	63.2%	25%
Choose not to say	1%	0%		1%	5%		0%	1%
Professional	18.86	19.26	15.50	12.94	10.86	12.05	12.96	17.5
experience Mean (SD)	(11.4)	(12.88)	(11.41)	(8.29)	(6.86)	(8.84)	(6.95)	(11.03)
Location of school								
Cities	97%	66.67%	93%	93%	78.41%	59.4%	80.5%	84.11%
Villages	1%	3.92%	7%	7%	10.23%	27.4%	19.5%	1.87%
sub-urban regions	2%	29.41%	0%	0%	11.36%	13.2%		11.21%
no answers		0%						2.8%
Type of school								
State-owned	39%	43.14%	46%	46%	100%	85.4%	100%	100%
Private	61%	48.04%	54%	54%		14.2%	0%	0%
Financial situation								
Very bad	0%	1.93%	1%	1%	0%	1.9%	1.1%	0%
Bad	5%	9.61%	9%	9%	4.5%	17.0%	12.6%	2.80%
Acceptable	73%	43.27%	39%	39%	27.3%	45.3%	54%	53.27%
Good	19%	40.39%	47%	47%	42.1%	32.1%	29.9%	34.58%
Very Good	3%	4.80%	4%	4%	26.1%	3.8%	2.3%	9.35%

## Results

As mentioned in the introductory section, an appropriate attitude towards new media is crucial for the effective and frequent use of ICT by teachers. Therefore, teachers were asked first to assess ICT in terms of learning and teaching processes. From the data collected, it can be concluded that the vast majority of teachers in each country like to use new technologies. To a slightly lesser extent, the teachers claim that new technologies have positively changed our lives. The most sceptical in this respect are the educators from Bolivia, while a very moderate position is taken by the educators from Poland. Those from Bolivia and Poland are also a little more sceptical about saying that it is now essential for us to use ICT in our learning and teaching. Teachers from Finland and Poland are a little more sceptical about the educational use of websites than teachers from other countries. It is noticeable, however, that respondents from each country are rather sceptical about the uncritical assessment of new media. Teachers from Uruguay are the most determined to assess the juxtaposition of digital and analogue methods. Educators from the Dominican Republic, Brazil, Turkey, Ecuador and Uruguay are much more likely to point to the positive impact new technology has on learning processes and motivation than teachers from other countries. The most divided position is held by educators from Bolivia, Poland and Finland. As can be seen in Table 2, the teaching staff from each country is not a homogeneous group when we consider the variable Attitude to new media.

**Table 2:** Descriptive statistics for indicators of the Attitude to new media variable

		I like to use digital technology	Digital technologies have positively changed our lives	It is necessary to use digital technologies in the process of learning and teaching	Web sites are useful for teaching and learning	Digital teaching aids are better than physical teaching aids on improving learning	The use of digital technologies by the teacher has a positive impact on student learning	The use of digital technologies by the teacher has a positive effect on student motivation
Country	N	Mean (SD)	Mean (SD)	Mean (SD)	Mean (SD)	Mean (SD)	Mean (SD)	Mean (SD)
Bolivia	154	4.136 (0.893)	3.234 (0.891)	3.815 (0.941)	3.980 (0.801)	2.869 (0.964)	3.636 (0.862)	3.773 (0.836)
Brazil	102	4.402 (0.649)	4.020 (0.832)	4.118 (0.812)	4.088 (0.646)	3.225 (1.160)	4.078 (0.740)	4.078 (0.754)
Dominican Republic	98	4.429 (1.005)	4.112 (1.004)	4.265 (1.061)	4.276 (1.003)	3.776 (1.162)	4.235 (1.003)	4.306 (0.957)
Ecuador	141	4.248 (1.063)	4.085 (1.112)	4.078 (1.089)	4.270 (1.062)	3.454 (1.025)	4.007 (1.045)	3.993 (1.066)
Finland	88	4.114 (0.988)	3.966 (0.928)	4.057 (0.987)	3.761 (1.124)	3.557 (0.869)	3.625 (1.158)	3.682 (0.891)
Poland	96	3.875 (0.798)	3.646 (0.894)	3.917 (0.879)	3.844 (0.850)	3.104 (0.840)	3.594 (0.734)	3.573 (0.778)
Turkey	87	4.218 (0.970)	3.908 (0.972)	4.345 (0.860)	4.011 (0.994)	3.069 (1.228)	4.287 (0.926)	4.184 (1.006)
Uruguay	107	4.421 (0.765)	3.916 (0.791)	4.009 (0.927)	4.047 (0.817)	2.748 (0.962)	3.944 (0.811)	3.907 (0.853)
		F = 4.437***	F = 13.255***	F = 3.582***	F = 4.094***	F = 12.640***	F = 9.357***	F = 7.701***

**Fig. 1:**
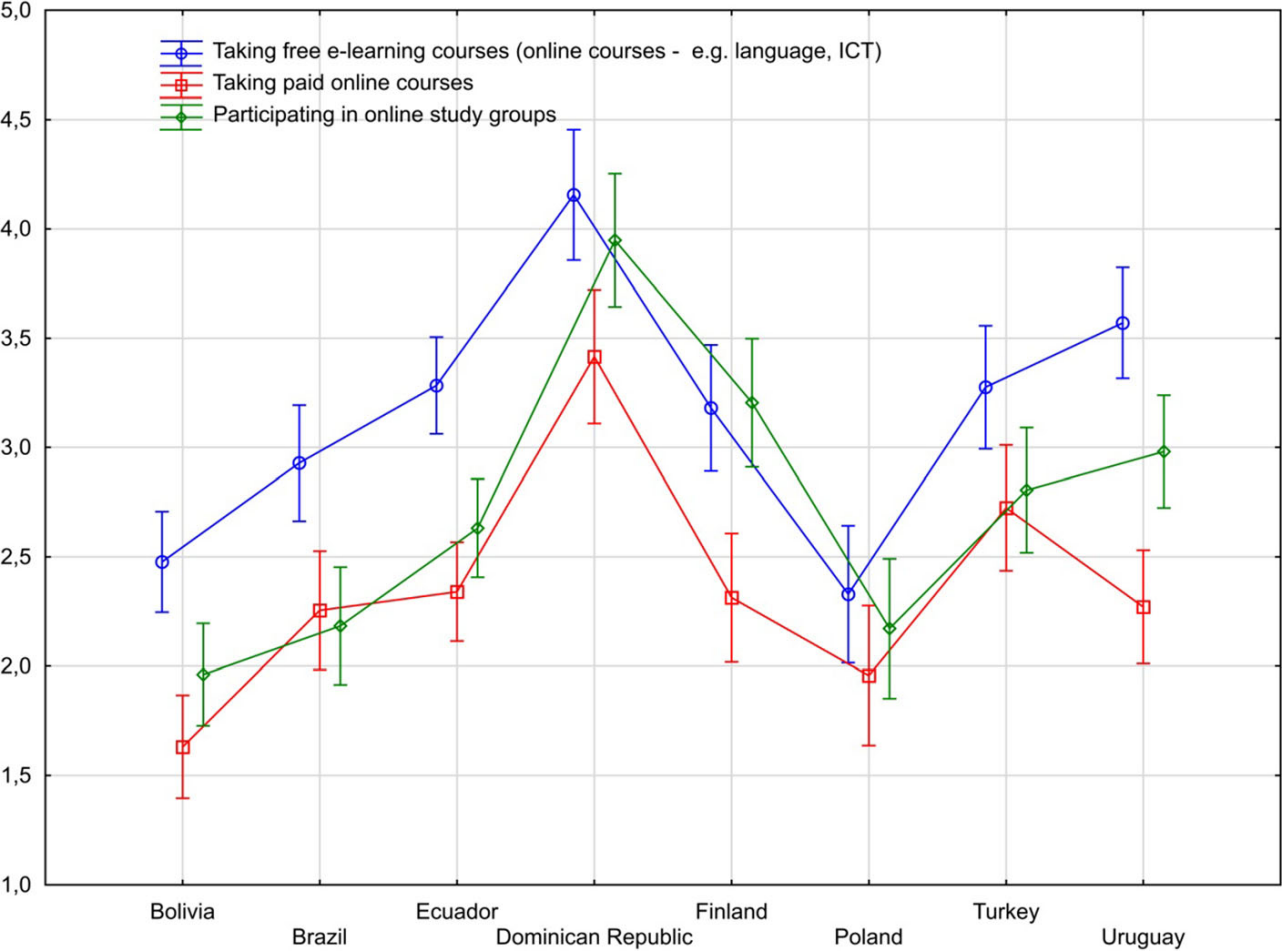
e-Learning experience among teachers from selected EU and LAC countries

Today, due to the epidemiological situation around COVID-19, e-learning has become a fundamental learning environment. This is a global trend that can be seen in the vast majority of countries around the world. The data presented in this text were collected in 2019, just before the onset of the pandemic that transformed education in both Europe and Latin America. The data collected is therefore, in a sense, historical, but it is worth showing how often e-learning solutions were used by teachers in individual countries before the pandemic. The results of comparative studies show that teachers from the Dominican Republic most often sought to improve their knowledge through paid and free courses, though this was rarely the case in Bolivia. Free online courses are much more popular than access to commercial platforms (there is no exception). In the European research sample, teachers from Poland used various forms of distance learning least frequently. A detailed comparison is available in Fig. 1.

Analysing the similarities and differences between the countries studied, it can be noted that the active use of new media correlates positively with the attitude towards new media. This correlation is visible among all LAC and EU teachers. For example, the more the new technologies are assessed as being a valuable tool in education, the more extensively do teachers use ICT. In many countries, attitudes towards new technologies are also positively correlated with the extensive use of e-learning (except for two European countries and Brazil). In two countries, it can also be observed that older teachers have less emphasis on the positive aspects of ICT use in education (Brazil, Dominican Republic).

It is interesting that the extent of the use of ICT in private life is positively correlated with the use of various forms of e-learning. Such a correlation only has a different correlative power depending on the country. The strongest correlation is observed in Brazil. There is also a generally strong correlation between the self-assessment of digital competence and the extent to which ICT is used. People who rate their digital competence highly also declare that they use ICT for private purposes much more extensively. A comparison of the correlations between the four variables (average values from the variables) and age as well as subjective evaluation of wealth is presented in Table 3. Spearman’s Correlations (a non-parametric test) was used in the calculations due to the distribution of indicators. The values presented in the table below show only the co-occurrence of variables.

**Table 3:** Spearman’s Correlations - comparison of the relationships between variables in LAC and EU countries

	Bolivia	Brazil	Dominican, Republic	Ecuador	Finland	Poland	Turkey	Uruguay
Attitude to new media - New media usage style	0.310***	0.205*	0.261**	0.146	0.303**	0.258*	0.399***	0.267**
Attitude to new media - Experiences with e-learning	0.174*	0.017	0.436***	0.205*	0.068	0.088	0.465***	0.389***
Attitude to new media - Self-evaluation of digital competence	0.249**	0.196*	0.249*	0.144	0.107	0.171	0.609***	0.305**
Attitude to new media - Age	−0.077	−0.289**	−0.229*	−0.171*	0.104	0.021	0.014	0.051
Attitude to new media - Financial situation	−0.088	0.130	−0.071	−0.225**	−0.227*	0.058	0.295**	0.009
New media usage style - Experiences with e-learning	0.352***	0.547***	0.446***	0.585***	0.189	0.150	0.633***	0.345***
New media usage style - Self-evaluation of digital competence	0.522***	0.613***	0.565***	0.524***	0.274**	0.319**	0.495***	0.486***
New media usage style - Age	0.049	−0.386***	−0.212*	−0.353***	0.011	−0.398**	0.119	0.001
New media usage style - Financial situation	−0.004	0.208*	−0.008	0.006	0.018	−0.012	0.129	0.070
Experiences with e-learning - Self-evaluation of digital competence	0.233**	0.406***	0.337***	0.405***	0.149	0.144	0.550***	0.329***
Experiences with e-learning - Age	−0.039	−0.163	−0.156	−0.266**	0.202	−0.047	−0.069	−0.021
Experiences with e-learning - Financial situation	−0.017	0.091	0.078	−0.142	0.049	0.004	0.234*	−0.129
Self-evaluation of digital competence - Age	−0.103	−0.344***	−0.186	−0.356***	0.042	−0.080	0.001	0.047
Self-evaluation of digital competence - Financial situation	−0.059	0.424***	−0.142	0.030	0.006	0.147	0.179	−0.123
Age - Financial situation	0.009	−0.022	0.044	0.092	0.059	−0.076	−0.043	0.213*

* *p* < .05,** *p* < .01,*** p < .001

**Fig. 2:**
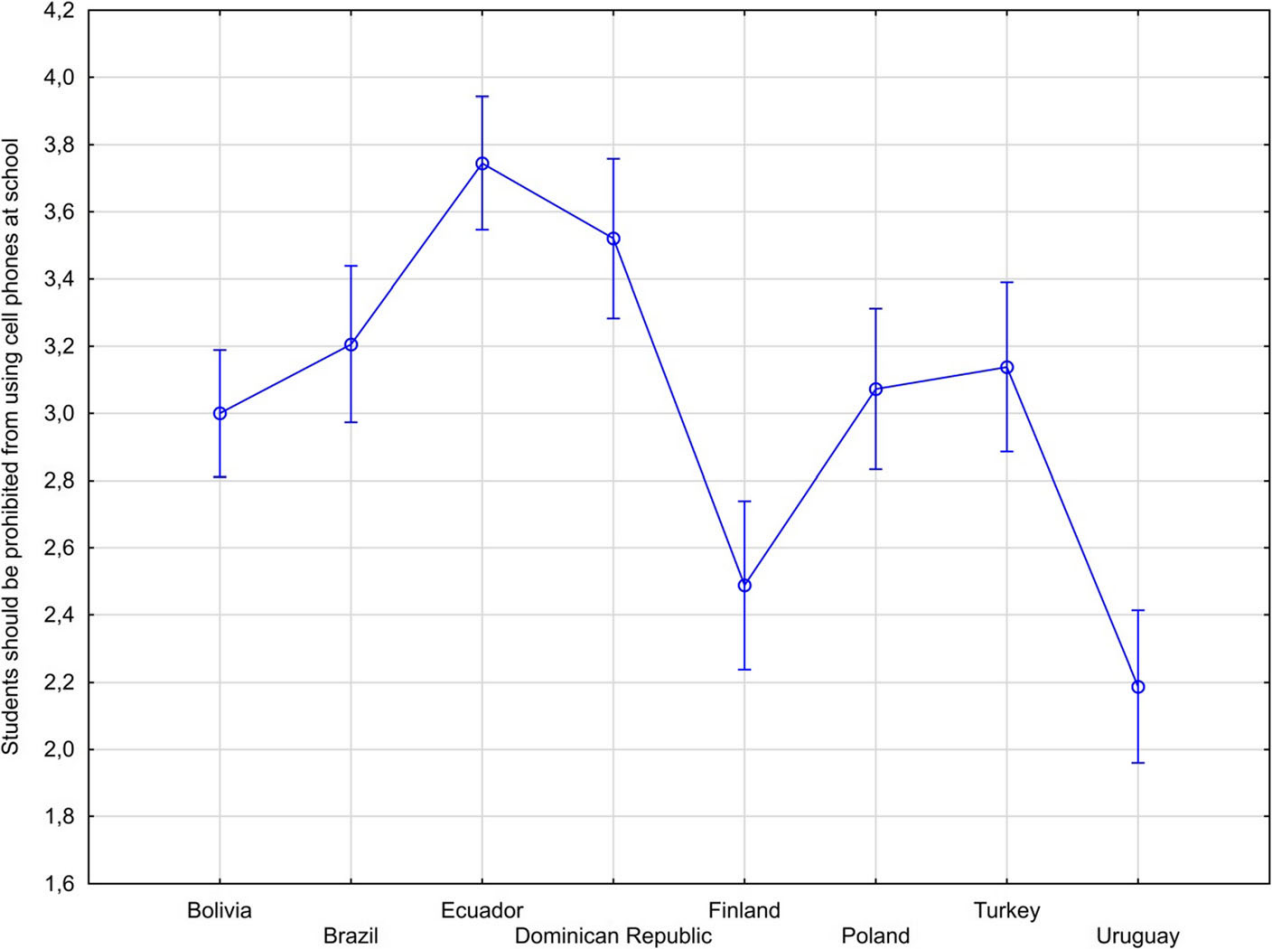
Should the use of telephones in schools be prohibited? (1-permitted, 5- forbidden)

The discussion on the use of mobile phones in education is an issue that has for some time generated mixed reactions among teachers, school management, parents and students. Therefore, as part of the comparative study, it was decided to ask educational staff about their consent regarding the general use of smartphones in schools. Based on the data collected it was noted that the most liberal answers are typical for teachers in Uruguay (AVG = 2.187, SD = 1.091) and Finland (AVG = 2.489, SD = 1.083). The most restrictive approach is represented by teachers from Ecuador (AVG = 3.745, SD = 1.105) and the Dominican Republic (AVG = 3.520; SD = 1.294). The standard deviation (SD) in all countries is similar (range between 1.09 and 1.33). A detailed comparative visualization is presented in Fig. 2.

Given the similarities and differences, we can say that teachers from Latin American countries are more restrictive about using smartphones in school. The exception is Uruguay, and this difference may be accounted for by the extensive process of digitisation of schools in that country. Similarly, in Europe, the most liberal approach is seen among the teachers from Finland, compared to similar statements made by teachers from Turkey and Poland. Detailed differences between countries are presented in Table 4.

**Table 4:** Differences between countries regarding restricting the use of mobile phones (Post Hoc Comparisons)

			95% CI for Mean Difference				
		Mean Difference	Lower	Upper	SE	t	Cohen’s d
Bolivia	Brazil	−0.206	−0.670	0.258	0.153	−1.349	−0.167
	Dominican, Republic	−0.520	−0.990	−0.051	0.154	−3.368	−0.428
	Ecuador	−0.745	−1.168	−0.321	0.139	−5.345	−0.656
	Finland	0.511	0.026	0.997	0.160	3.200	0.451
	Poland	−0.073	−0.545	0.399	0.155	−0.469	−0.062
	Turkey	−0.138	−0.625	0.349	0.160	−0.860	−0.112
	Uruguay	0.813	0.356	1.270	0.150	5.404	0.717
Brazil	Dominican, Republic	−0.315	−0.829	0.200	0.169	−1.857	−0.239
	Ecuador	−0.539	−1.012	−0.066	0.156	−3.463	−0.446
	Finland	0.717	0.188	1.246	0.174	4.118	0.585
	Poland	0.133	−0.384	0.650	0.170	0.781	0.105
	Turkey	0.068	−0.463	0.599	0.175	0.389	0.050
	Uruguay	1.019	0.516	1.522	0.166	6.151	0.837
Dominican, Republic	Ecuador	−0.224	−0.703	0.254	0.157	−1.425	−0.189
	Finland	1.032	0.498	1.566	0.176	5.869	0.861
	Poland	0.447	−0.075	0.970	0.172	2.603	0.361
	Turkey	0.382	−0.153	0.918	0.176	2.169	0.289
	Uruguay	1.333	0.825	1.842	0.167	7.967	1.118
Ecuador	Finland	1.256	0.762	1.750	0.163	7.723	1.146
	Poland	0.672	0.190	1.153	0.158	4.241	0.591
	Turkey	0.607	0.111	1.103	0.163	3.718	0.503
	Uruguay	1.558	1.091	2.024	0.153	10.150	1.418
Finland	Poland	−0.584	−1.121	−0.047	0.177	−3.307	−0.515
	Turkey	−0.649	−1.199	−0.099	0.181	−3.587	−0.529
	Uruguay	0.302	−0.222	0.825	0.172	1.751	0.277
Poland	Turkey	−0.065	−0.603	0.473	0.177	−0.367	−0.051
	Uruguay	0.886	0.375	1.397	0.168	5.265	0.781
Turkey	Uruguay	0.951	0.426	1.476	0.173	5.503	0.781

Given that attitudes towards the use of new technologies (including smartphones) are not a constant feature, that they also change under the influence of circumstances (such as the COVID-19 situation), the LAC and EU researchers decided to show the impact of factors such as attitudes towards new technologies, the use of new media, experiences with e-learning and the self-evaluation of digital competences in relation to the dependent variable, i.e. the use of smartphones in schools. A detailed analysis for each country is presented in Table 5.

**Table 5:** Power of predictors for prohibitions using mobile phones at schools

Variable	Bolivia	Brazil	Dominican, Republic	Ecuador	Finland	Poland	Turkey	Uruguay
	β	β	β	β	β	β	β	β
I like to use digital technologies	−0.007	−0.191	0.114	0.186	0.233	−0.218	−0.231	−0.215
Digital technologies have positively changed our lives	−0.014	0.030	−0.035	−0.071	0.032	−0.280	−0.076	−0.212
It is necessary to use digital technologies in the process of learning and teaching	−0.096	−0.137	0.398	0.022	−0.019	0.185	0.259	−0.019
Web sites are useful for teaching and learning	−0.208	−0.024	−0.179	−0.039	0.278*	−0.061	0.102	0.020
Digital teaching aids are better than physical teaching aids on improving learning	−0.060	0.576*	0.570**	0.135	0.255	0.043	0.197	0.159
The use of digital technologies by the teacher has a positive impact on student learning	0.031	0.382	−0.574	−0.054	−0.073	0.350	0.286	0.222
The use of digital technologies by the teacher has a positive effect on student motivation	−0.127	−0.202	0.213	−0.102	−0.120	−0.272	0.251	−0.028
The use of digital technologies by the teacher has a positive effect on student satisfaction	−0.064	−0.531	0.277	0.065	−0.143	−0.214	−0.380	−0.111
Publishing messages on Internet	0.238	0.109	0.176	−0.110	0.068	0.015	−0.010	−0.119
Consuming Internet streaming (eg. VOD)	0.050	−0.254	−0.132	−0.190	−0.124	0.007	0.031	−0.119
Creating video	−0.233	−0.150	−0.078	0.253*	0.546**	0.227	0.120	−0.240
Using a file sharing service	0.100	−0.113	0.459	0.143	−0.159	−0.386	0.067	−0.059
Participating as member of a group	−0.190	−0.109	−0.141	−0.117	0.106	0.192	−0.203	0.096
Accessing online services – e-government	−0.044	0.121	−0.347	0.060	−0.120	0.078	−0.129	0.018
Buying/Selling goods	−0.105	−0.190	0.133	−0.128	−0.205	−0.130	0.151	0.140
Leisure	−0.247	0.473	−0.121	0.004	−0.078	−0.168	−0.291	−0.185
Study in an obligatory online course in my career or in my postgraduate studies	0.087	−0.277	0.067	−0.068	0.388*	0.094	0.260	0.016
Taking free e-learning courses (online courses - e.g. language, ICT)	0.212	0.483	−0.338	0.011	−0.069	0.440	−0.192	0.009
Taking paid online courses	0.131	−0.012	−0.025	−0.006	−0.123	0.016	−0.137	−0.044
Participating in online study groups	−0.090	−0.244	0.202	−0.015	−0.268	−0.161	0.077	−0.118
Using the text editor (e.g. Word, writer)	−0.072	0.016	0.241	−0.153	0.061	−0.147	−0.058	−0.019
Using the Spreadsheet (e.g. Excel, Calc)	−0.050	−0.170	0.014	0.053	−0.078	0.228	−0.093	−0.160
Using the presentation program (e.g. Power Point, impress)	0.036	0.132	0.058	0.216	−0.147	−0.196	0.311	0.195
Using the graphic program (e.g. Picasa, Gimp)	0.200	0.253	0.034	0.089	0.250	0.000	−0.234	0.151
Knowledge about the dangers of the digital world (e.g. cyberbullying, Internet addiction, sexting)	0.083	0.081	−0.342	−0.289*	0.127	0.099	−0.203	0.199
Age	−0.017	0.143	−0.125	−0.294**	0.232	0.087	0.151	0.036
Financial situation	0.194	−0.134	0.152	0.229*	0.061	−0.131	−0.031	0.037
	R = 0.647 R^2^ = 0.419 F(270.48) = 10.286 *p* < 0.219	R = 0.605 R^2^ = 0.366F(270.69) = 10.4781 *p* < 0.098	R = 0.770 R^2^ = 0.593 F(270.44) = 20.377 *p* < 0.005	R = 0.526R^2^ = 0. 276F(27.113) = 1.6015 *p* < 0.046	R = 0.814 R^2^ = 0.664 F(270.55) = 40.028 *p* < 0.000	R = 0.697 R^2^ = 0.486F(270.39) = 10.370 *p* < 0.18119	R = 0.605 R^2^ = 0.367 F(270.59) = 10.2680 *p* < 0.220	R = 0.613 R^2^ = 0.376 F(270.79) = 10.768 *p* < 0.026

β – Standardized coefficient;*p < 0.05,** < 0.01.

Multilinear regression analysis was used for the calculations. In order to simplify the presentation of the data, Table 5 shows: β - Standardized coefficient and confidence level (p) as well as for each country the coefficient of determination (R2), which explains the possibility of a percentage generalisation in the available sample. In most countries, the sample collected allows for an explanation of the likelihood of increasing negative attitudes to smartphones in school between 33% and 66%. From the data collected, it was noted that in most countries attitudes towards new technologies in educational contexts, new media usage patterns, and self-evaluation of digital competences do not change. The attitude towards smartphones is therefore a more permanent construct. It is, however, encouraging that there is a trend among teachers in Brazil and the Dominican Republic that, with an increase in restrictive attitudes towards smart phones, there is a minimal (weak) increase in the positive rating of digital teaching aids compared to analogue counterparts. Teachers from Finland also stand out from other countries due to the increase in restrictiveness, which is not necessarily linked to a constant assessment of the use of new media in education. It is also worth noting the case of Ecuador, where the declared knowledge about the dangers of the digital world decreases with increasing restriction.

## Discussion

First, it is important to point out the weakness of this study in terms of the samples considered. Not only are they small in number, but most importantly, the samples are not statistically representative of the whole population of teachers in each country. Also, these data were generated by a self-declaration of competencies. However, considering the lack of similar comparative studies, these findings suggest interesting avenues for further research in comparing EU and LAC teachers with larger, representative samples.

The first result mentioned is that the vast majority of teachers like to use digital technologies and consider that digital technologies have positively changed our lives. This is a fundamental point of departure before any further analysis: where do teachers stand in reference to technology; is there rejection or acceptance? Is it part of a teacher’s personal life? Has it been part of their own professional development path?

In this study, attitudes towards new media were surveyed by questioning teachers’ preference, their perception of technology as representing a positive change, their opinion on the usefulness of technology in teaching and learning, whether technology had a positive effect on motivation, and whether technology was necessary for learning (Machmud and Abdulah 2017). There was also a question asking teachers to consider whether digital teaching aids were better than physical or analogue ones. The results highlight teachers’ positive response to technology. Observing responses by country leads to the question of which variables may be influencing this attitude. The literature on the topic traces such influences back to a variety of approaches that study how individuals adopt technology: the theoretical framework based on the technology acceptance model (TAM), or between perceived usefulness and behavioural intention. Some studies integrate other variables into the model, accounting for specific contextual conditions, such as quality of life (Tarhini et al. 2017a, 2017b) or cultural variables such as power distance or individualism- collectivism (Huang et al. 2019).

Taking into account these perspectives and observing the responses collected in this study some questions emerge: do differences among countries relate to cultural differences in terms of values or do they respond to contextual infrastructural variables such as the availability of high quality technology and good connectivity? The idea that specific cultural variables be considered internationally, would be an interesting line of research to pursue. Clearly, and with all the shortcomings of the samples in mind, differences among countries do not reflect a continental divide. Would such differences be the result of national achievements in terms of overcoming the digital divide or is there instead a response to specific educational policies towards teacher education and development?

Not surprisingly, some correlations are found between the extent to which technology is used in private life and participation in e-learning experiences. Also, between the extensive use of technologies and digital competency. This result adds up to the notion that digital skills among adults and the private use of technology are closely related. A conceptual framework developed by Wicht, Lechner and Reder, for the understanding of ICT use as a prerequisite for digital skills development, concludes that “digital skills do not emerge in a vacuum but are strongly contingent on individuals’ ICT use at work and in everyday life. That is, adults acquire digital skills largely through ‘learning by doing’“. (Wicht et al. 2019). Competency development first requires access, then practice.

As for digital literacy in this self reported survey, it seems to be more focused on technology and the skills required to use it, than on the reflective use it might require (List et al. 2020). The general survey question requests a self-reported level of skill, but it does not necessarily consider other dimensions of literacy that involve higher order thinking skills or the development of a critical attitude towards the *what*, *why* and *how* of the implementation of technology in education (Morales 2018; Martínez et al. 2017).

Considering the correlations that the study has highlighted, it is reasonable to see a relationship between affluence and digital competency. Studies of the digital divide confirm that the traditionally disadvantaged have fewer chances to access high speed connections or appropriate devices, and this is clearly an obstacle to the initiation of the process of literacy development. (Helsper 2019). More affluent teachers are more likely to have access to good connections and to devices that allow them to develop more extensive and diverse uses of technology, and consequently to benefit more from the tools available.

At this point, it is relevant to consider whether this generally positive attitude found towards integrating technology into the teaching and learning processes would be reflected in teaching practices. The adoption of technology relates not only to teachers’ attitudes, willingness and motivation (Sharma and Srivastava 2019), it also implies a process that goes from technology as a kind of support that does not necessarily affect teaching, to the actual integration of technology into the structure of the classroom and the behaviours found within (Nicolle and Lou 2008). It is advisable that future studies explore the practical uses of digital technologies in actual teaching.

One of the most revealing results of this study refers to how teachers perceive the use of smartphones in the classroom, their potential as a learning tool and what to do about them. The respondents were asked if students should be allowed to use smartphones in the class. Numerous studies have shown that teachers’ perception of this issue is not homogenous. Among the positive aspects of smartphone use, there is the fact that it helps to find up-to-date information, it increases searching and learning skills, and provides learning opportunities anytime, anywhere (Wali and Omaid 2020). Other studies stress that the portability of smartphones allows everything from written notes to whole lectures to be captured through the smartphone’s camera. (Anshari et al. 2017). Studies carried out with higher education students reveal that the appropriate use of smartphones increases connectedness and out-of-class involvement (Liu et al. 2016). On the other hand, some of the facts that hold teachers back from encouraging the use of smartphones in class are associated with disruptions due mainly to disconnection from face to face activities, cheating, and the negative impact of text messaging services on students’ writing skills (Wali and Omaid 2020).

In terms then of the use of smartphones, the most liberal answers were provided by teachers in Uruguay and Finland, and the most restrictive responses came from teachers in Ecuador and the Dominican Republic (see Fig. 2). The question must be posed as to whether Uruguayan connectivity standards or Finnish innovative educational approaches play a role in the development of the teachers’ positive attitudes. Future studies would have to explore these variables. But one of the most interesting challenges underlying this dilemma is understanding youth culture and the relevance that smartphones have in the “mobile lifestyle” (Vanden Abeele 2016). As researchers, this means posing questions that place technology-associated practices into a broader cultural context that contains both teachers and students with diverse technology-associated experiences.

Another result that raises interesting questions and suggests research possibilities in this study is connected to teachers’ professional development with e-learning experiences. The teachers were asked how frequently they had used the Internet for e-learning activities. The results show that free online courses are the most widely used tool. Further studies should investigate whether this is a response to the lack of face-to-face possibilities within reach in the countries of origin to specialize or pursue postgraduate courses, or if it is more connected to the flexibility offered by such programs in terms of time and autonomy for self-learning, or to other more innovative mechanisms that allow the teachers to participate in professional learning communities. Studies show that MOOCS have a high potential to deliver large scale, self-regulated instruction on digital skills or open educational resources (Wambugu 2018). They are also a way to overcome time constraints and resource shortage, which are signs of a promising outlook that researchers should further study (Ji and Cao 2016; Misra 2018).

The recent Covid 19-related compulsory shift from face-to-face teaching and learning to emergency remote teaching has confirmed the value of teacher training and professional development programs that allow teachers to display an autonomous, literate, contextualized, and appropriate integration of technologies into their teaching practices. Research results like those displayed by SELI provide valuable insights into what has become not only relevant but necessary and urgent as an object of study.

In this context, how should techno-optimism be understood? Is technology simply a neutral tool, a means to an end that teachers adopt because it is handy? Does technology have its own agency, following an inevitable path? Or should teachers go beyond this instrumental view of technology and familiarize themselves with it more fully, reflect upon its use, and make critical choices? (Adell 2018) These are questions worth asking, but the answers will have to be seen through the lens of further research. As Westera comments, “At the symbolic level, educational technology should strive to go ‘beyond functionality and efficiency’ and pursue added values that make education interesting, tough, important, intriguing and the like” (Westera 2005, p.35).

Would techno-optimism imply that teachers believe technology is going to “solve every problem”? This view would certainly reduce a demanding task to simple blind adherence. When it comes to integrating technology, each social group will develop a different process, dependent not only on the availability of technology, but also on cultural variables and institutional contexts. They will depend largely on the adoption strategies developed by educational institutions, determined by educational policies, which might vary greatly from country to country.

### Limitations of the research and new directions

This study was limited in its ability to draw adequate random samples, and samples representative of the population from the teachers in several countries within the study from which the outcome of the study should be generalized. Therefore, this study sample is not representative of the population of teachers and our results are not generalizable.

Owing to the small sample of the research data, this study may be limited in terms of supporting replication. The replication of results in studies such as this is supposed to support both generalizability and the internal validity of similar existing research. However, due to sample variability and inadequacy of the data for making a generalizable sample, the outcome of this study may result in a potential bias and confusion, which are practically visible in this study. However, the rationale for advancing this study was the heterogeneity of the population sampled and uses of knowledge claims in essential cases of the application of technology in teaching practices by teachers across the EU and LAC contexts of this study.

The problem of generalizability in this study occurs as a result of many factors, including the inadequacy of the samples obtained, this being occasioned by low response rates among teachers across the study areas; the locations and cultural settings of the participants, as many teachers were inaccessible during the period of data collection due to certain engagements. Moreover, the Trans-Atlantic nature of this research makes it quite difficult to access a good sample of teachers.

To address the issue of the generalizability of the study results in future research, there is a need to replicate the findings in this study among a larger sample of teachers in settings across the EU and LAC regions and other countries, to expand the sample to generalize within other related empirical settings such as pre-service teachers in order to cover a larger population, and to conduct a comparative case study in several academic institutions that will support other means of obtaining inferences and extrapolating beyond the context of teachers’ perspectives to more qualitative methodological approaches.

Applying the current assessment of competences, based on the self-declaration of personal opinion and the teachers’ experiences, constitutes a weak point in the measurement of the detailed and baseline attitudes towards the technological attributes. The benefits of the teachers’ self-declarations are many, including the fact that the quantitative data collection through the questionnaire was a relatively simple, quick, and low cost approach that suited our needs given the wide geographical scope of the research, encompassing both areas of the EU and of LAC. However, several reasons led to the self-declaration threatening the validity and reliability of the measurements obtained in this study. For example, the huge variability of the respondents’ characteristics, such as culture, geographical location, and national economic outlook may have impacted on the validity of our measurements.

In future research, we will conduct a more comprehensive study about teachers’ technological attitudes and professional practices. For example, comparative research could be carried out between several EU and LAC countries that covers larger samples to obtain much needed insights into the teachers’ vantage points of the application of novel technological media in education.

### Summary

The research shows that teachers, in general, tend to be techno-optimists. However, different approaches exist among countries, even within the same region, as is the case with Bolivia compared to the other LAC countries. Overall, the positive attitude teachers have demonstrated towards technology has been instrumental in facing, with relative success, the consequences of the COVID-19 pandemic in education systems worldwide. These observations are even more significant if we consider the fact that this work was carried out before the coronavirus spread across the world and became a pandemic.

It is evident what the role is that ICTs play in society, and the crucial importance ICTs have in the teaching-learning process. Similarly, it is worth noting how teachers have gradually moved towards the use of ICTs in the classroom, albeit slowly, but in an incremental fashion.

A digital divide exists between European countries (e.g., Finland and Poland) and Turkey versus LAC countries (e.g., Bolivia, Brazil, the Dominican Republic, Ecuador and Uruguay), as a result of the technological prowess and increased development of EU countries compared to the poor quality of investment by LAC governments to support education. This notwithstanding, teachers are increasingly using ICTs and have understood the increasing importance of technology in recent times. In this regard, we see an agreement in the positive outlook of ICTs in EU and LAC countries (Tomczyk and Oyelere 2019; Tomczyk et al. 2019).

In order to effectively commit to the use of ICTs in education, it is necessary to start with a diagnosis of the technological elements and of the relevant competencies (Galustyan 2020; Stošić and Stošić 2015; Zerkina 2018). This baseline will allow for the identification of current strengths and areas ripe for improvement, followed by the establishment of training strategies for all of the stakeholders in the process. Therefore, these elements represent the minimum criteria necessary to guarantee a successful ICT-based teaching strategy and the development of meaningful learning.

Even though COVID-19 has accelerated the process by which teachers have integrated ICT into their work, it is still necessary to continue supporting its use so that teachers are able to absorb ICTs as a regular aspect of their teaching-learning ecosystem, and not just as an emergency measure. However, in some countries, especially in Latin America and the Caribbean (e.g., Ecuador, Bolivia, and the Dominican Republic), it may be necessary to wait for the emergence of a new generation of teachers, who can fully adopt ICTs as an intrinsic part of their teaching activities.

## Appendix

**Table 6:** Factor Loadings

	Factor 1	Factor 2	Factor 3	Factor 4	Uniqueness
I like to use digital technologies	0.678				0.500
Digital technologies have positively changed our lives	0.701				0.495
It is necessary to use digital technologies in the process of learning and teaching	0.773				0.383
Web sites are useful for teaching and learning	0.694				0.527
Digital teaching aids are better than physical teaching aids on improving learning	0.551				0.672
The use of digital technologies by the teacher has a positive impact on student learning	0.844				0.306
The use of digital technologies by the teacher has a positive effect on student motivation	0.841				0.315
The use of digital technologies by the teacher has a positive effect on student satisfaction	0.793				0.390
Publishing messages on Internet		0.637			0.558
Consuming Internet streaming (eg. VOD)		0.688			0.538
Creating video					0.577
Using a file sharing service		0.765			0.398
Participating as member of a group		0.760			0.453
Accessing online services – e-government		0.536			0.487
Buying/Selling goods		0.408			0.623
Leisure		0.607			0.633
Study in an obligatory online course in my career or in my postgraduate studies				0.714	0.501
Taking free e-learning courses (online courses - e.g. language, ICT)				0.774	0.365
Taking paid online courses				0.847	0.382
Participating in online study groups				0.732	0.419
Using the text editor (e.g. Word, writer)			0.745		0.395
Using the Spreadsheet (e.g. Excel, Calc)			0.885		0.314
Using the presentation program (e.g. Power Point, impress)			0.805		0.324
Using the graphic program (e.g. Picasa, Gimp)			0.684		0.492
Knowledge about the dangers of the digital world (e.g. cyberbullying, Internet addiction, sexting)			0.553		0.663
Smartphones prohibition					0.952

*Note.* Applied rotation method is promax
